# A CT Reconstruction Algorithm Based on L_1/2_ Regularization

**DOI:** 10.1155/2014/862910

**Published:** 2014-04-16

**Authors:** Mianyi Chen, Deling Mi, Peng He, Luzhen Deng, Biao Wei

**Affiliations:** The Key Lab of Optoelectronic Technology and Systems of the Education Ministry of China, Chongqing University, Chongqing 400044, China

## Abstract

Computed tomography (CT) reconstruction with low radiation dose is a significant research point in current medical CT field. Compressed sensing has shown great potential reconstruct high-quality CT images from few-view or sparse-view data. In this paper, we use the sparser L_1/2_ regularization operator to replace the traditional L_1_ regularization and combine the Split Bregman method to reconstruct CT images, which has good unbiasedness and can accelerate iterative convergence. In the reconstruction experiments with simulation and real projection data, we analyze the quality of reconstructed images using different reconstruction methods in different projection angles and iteration numbers. Compared with algebraic reconstruction technique (ART) and total variance (TV) based approaches, the proposed reconstruction algorithm can not only get better images with higher quality from few-view data but also need less iteration numbers.

## 1. Introduction

Since computed tomography (CT) technique was born in 1973, CT has been widely applied in medical diagnose, industrial nondestructive detection, and so forth [1]. In medical CT field, CT reconstruction with low radiation dose is a significant research problem, which needs the reconstruction of high-quality CT images from few-view or sparse-view data. Recently, compressed sensing (CS) [2] theory has been applied in CT image reconstruction; it is possible to reconstruct high-quality images from few-view data under the frame of CS. In CS theory, sparse signal is often regularized with L_*p*_(0 ≤ *p* ≤ 1) regularization; L_0_ regularization is the sparest and most ideal regularization norm (L_0_ regularization denotes the number of nonzero signal elements). However, L_0_ regularization is susceptible to noise interference and it is difficult to solve equations, and L_1_ regularization is usually used as the regularization operator. Theoretically, if regularization is closer to L_0_ regularization could get higher-quality CT images in CT reconstruction.

Recently, Xu et al. proposed L_1/2_ regularization [3] and a soft thresholding algorithm [4], and Xu and Wang proposed a hybrid soft thresholding algorithm [5]. L_1/2_ regularization has some theoretical properties, such as unbiasedness, sparse regularization, and Oracle. It is easier to produce sparser solution compared with the current  L_1_  regular operator used in CT reconstruction. Theoretically, CT reconstruction method based on L_1/2_ regularization will reconstruct higher-quality CT images with few-view data.

On solving L_1_ regularization problem, Goldstein and Osher proposed Split Bregman method [6] which is derived from Bregman iterative algorithm [7]. Split Bregman method uses an intermediate variable to split L_1_ regularization and L_2_  regularization into two equations; L_2_  regularization equation can be solved by gradient descent method, and L_1_ regularization equation can be solved by thresholding algorithm. Split Bregman method can accelerate iterative convergence and produce better results. Based on Split Bregman method, Vandeghinste et al. proposed Split Bregman-based sparse-view CT reconstruction [8] and iterative CT reconstruction using shearlet-based regularization [9]. Chu et al. proposed multienergy CT reconstruction based on low rank and sparsity with the Split Bregman method (MLRSS) [10]. Chang et al. proposed a few-view reweighted sparsity hunting (FRESH) method for CT image reconstruction [11].

In this paper, we propose a CT reconstruction algorithm based on L_1/2_ regularization and Split Bregman method. In the following section, L_1/2_ regularization, Split Bregman method, and the proposed algorithm will be introduced. In the third section, we will use the proposed algorithm to analyze numerical phantom and real projection data. In the last section, we will discuss relevant issues and conclude the paper.

## 2. Materials and Methods

### 2.1. L_1/2_ Regularization

Generally, CT reconstruction algorithm can be divided into analytic reconstruction algorithm and iterative reconstruction algorithm; the current typical analytic reconstruction algorithm is filter back-projection (FBP), and iterative reconstruction algorithm contains algebraic reconstruction technique (ART) [12], simultaneous algebraic reconstruction technique (SART) [13], expectation-maximization (EM) [14], and so forth. Mathematical model [15] of CT image reconstruction can be expressed as

(1) $$\begin{array}{c} Au=b, \end{array}$$

where **A** = (*a*_*ij*_) is the projection matrix, **b** = (*b* ^1^, *b* ^2^,…, *b* ^*M*^) ∈ *R* ^*M*^ is the projection data, and **u** = (*u*_1_, *u*_2_,…, *u*_*N*_) ∈ *R* ^*N*^ is the reconstruction image.

For sparse-view data, it is difficult to reconstruct high-quality images using the conventional CT image reconstruction algorithms, especially for analytic reconstruction algorithms which require high completeness of data. Meanwhile, there are also some artifacts in the reconstruction images using the conventional iterative reconstruction algorithms. In 2006, Donoho put forward the compressed sensing (CS) theory [2]; its main idea is that most of signals are sparse in the proper orthogonal transform domain, which means most of signal transformation coefficients are close to zero or equal to zero in orthogonal transformation, such as gradient transformation [16] and shearlet transformation [9]. In CS theory, an image can be reconstructed from a rather limited amount of data as long as an underlying image can be sparsely represented in an appropriate domain and determined from these data. CT sampling signal is a typical sparse signal; the x-ray attenuation coefficients of some regions of tested objects (i.e., human body) are similar or equal. Thus, CT reconstruction approaches based on compressed sensing can reconstruct high-quality CT images from sparse-view data.

CT reconstruction problem can be converted to a constrained optimization problem

(2) $$\begin{array}{c} \underset{u}{\min}\;E\left(u\right)\;\text{s}.\text{t}.\;\;Au=b, \end{array}$$

where *E*(*u*) is the regularization function, usually denoted by L_1_ norm of wavelet, gradient, and so forth. In order to simplify (2), we can use the penalty function method in the optimization method to convert it into an unconstraint optimization problem

(3) $$\begin{array}{c} \underset{u}{\min}\;E\left(u\right)+\lambda_{k}{||b-Au||}_{2}^{2}, \end{array}$$

where *λ*_*k*_ is the weight coefficient, *E*(*u*) can be denoted by total variation (TV) which is a research hotspot in current CT research field. TV method has been widely used in sparse-view and limited angle CT reconstruction [7].

In compressed sensing theory, L_0_ norm is the most ideal regularization norm, but it is difficult to solve equations with L_0_ norm, and L_0_ regularization is easily interfered by noise in CT reconstruction, so L_0_ norm is commonly replaced by L_1_ norm. Theoretically, using a regularization norm which is closer to L_0_ norm will reconstruct higher-quality CT images. The definitions of L_0_, L_1_, and L_1/2_ regularization norm are

(4) ||U||0=a,||U||11=∑i=1N|ui|,||U||1/21/2=∑i=1Nui,

where *U* = (*u*_1_, *u*_2_,…, *u*_*N*_) and *a* denotes the nonzero elements number in matrix *U*.

**Figure 1:**
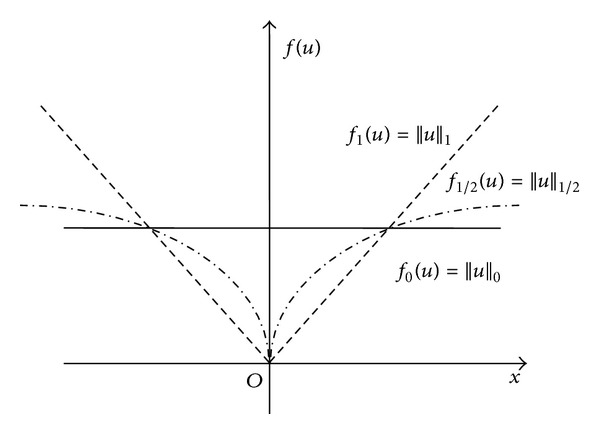
L_0_, L_1_, and L_1/2_ regularization norm. Solid line and dotted line represent L_0_ and L_1_ regularization norm, respectively; imaginary point line denotes L_1/2_ regularization norm.

As shown in Figure 1, L_1/2_ regularization norm is closer to L_0_ regularization norm than L_1_ regularization norm.

Xu et al. proposed a L_1/2_ regularization fast algorithm [4]; it can be expressed as

(5) $$\begin{array}{c} \underset{x}{\min}\left\{{||b-Au||}_{2}^{2}+\gamma {||u||}_{1/2}^{1/2}\right\}, \end{array}$$

where *γ* is the regularization coefficient.

If *u* is the optimal solution of (5), then the optimal condition of (5) will be denoted by

(6) $$\begin{array}{c} 0=A^{T}\left(Au-b\right)+\frac{\gamma }{2}\nabla \left({||u||}_{1/2}^{1/2}\right), \end{array}$$

where ∇(||*u*||_1/2_^1/2^) is the gradient of ||*u*||_1/2_^1/2^; multiplying coefficient *μ* and adding *u* at the both sides of (6), then we have

(7) $$\begin{array}{c} u+\mu A^{T}\left(b-Au\right)=u+\frac{\mu \gamma }{2}\nabla \left({||u||}_{1/2}^{1/2}\right). \end{array}$$

The definition of operator is

(8) $$\begin{array}{c} R_{\gamma ,1/2}={\left(I+\frac{\gamma }{2}\nabla \left({||u||}_{1/2}^{1/2}\right)\right)}^{-1}. \end{array}$$

Then the optimal solution *u* can be represented as

(9) u=(I+γ2∇(||u||1/21/2))−1(u+μAT(b−Au))=Rμγ,1/2(u+μAT(b−Au)),

where the operator is

(10) Rμγ,1/2=(fγ,1/2(u1),fγ,1/2(u2),…,fγ,1/2(uN))T,fγ,1/2(ui)=23ui(1+cos⁡⁡(2π3−23φγ(ui))),φγ(ui)=arccos⁡(γ8(|ui|3)−3/2).

Please see [4] for more proof details.

### 2.2. Split Bregman Method

In order to solve (3), Goldstein and Osher proposed Split Bregman method [6], using an intermediate variable to split L_1_ regularization and L_2_ regularization into two equations; L_2_ regularization equation can be solved by gradient descent method and L_1_ regularization equation can be solved by thresholding algorithm. Then the unconstrained optimal problem of (3) can be converted into

(11) $$\begin{array}{c} u=\underset{u}{\text{argmin}}{||\Phi \left(u\right)||}_{1}^{1}+\lambda {||b-Au||}_{2}^{2}, \end{array}$$

where Φ is the sparse transform and the common used sparse transform contains gradient, wavelet, shearlet, and so forth.

Using an intermediate variable *d* = Φ(*u*), (11) can be converted into

(12) $$\begin{array}{c} u^{k+1}=\underset{u}{\text{argmin}}{||d||}_{1}^{1}+\lambda {||b-Au||}_{2}^{2}+\mu {||d-\Phi \left(u\right)||}_{2}^{2}, \end{array}$$

where *μ* is the coefficient. Then (12) can be converted into two unconstrained optimal Bregman problems; it can be expressed as

(13) (uk+1,dk+1)=argminu||d||11+λ||b−Au||22+μ||d−Φ(u)−bk||22,

(14) $$\begin{array}{c} b^{k+1}=b^{k}+\left(\Phi \left(u^{k+1}\right)-d^{k+1}\right). \end{array}$$

Equation (13) can split into two equations:

(15) uk+1=argminuλ||b−Au||22+μ||d−Φ(u)−bk||22,

(16) dk+1=argminu||d||11+μ||d−Φ(u)−bk||22.

There are several advantages of Split Bregman method. Firstly, Split Bregman method can accelerate iterative convergence and calculate better results. Secondly, Split Bregman method can be widely used in CT reconstruction; it can not only solve L_1_ regularization problem but also solve other regularization problems.

### 2.3. CT Reconstruction Algorithm Based on L_1/2_ Regularization

According to aforementioned methods, we propose a CT reconstruction algorithm based on L_1/2_ regularization, where L_1/2_ norm is used as the regularization norm and gradient as the sparse conversion; then (11) can be expressed as

(17) $$\begin{array}{c} u=\underset{u}{\text{argmin}}{||\nabla u||}_{1/2}^{1/2}+\lambda {||b-Au||}_{2}^{2}. \end{array}$$

Combine with Split Bregman method to solve (17) as follows.

**Step 1**

One has

(18) $$\begin{array}{c} u^{k+1}=\underset{u}{\text{argmin}}\lambda {||b-Au||}_{2}^{2}+\mu {||d^{k}-\nabla u-b^{k}||}_{2}^{2}. \end{array}$$

**Step 2**

One has

(19) $$\begin{array}{c} d^{k+1}=\underset{d}{\min}{||d||}_{1/2}^{1/2}+\mu {||d-\nabla u^{k+1}-b^{k}||}_{2}^{2}. \end{array}$$

**Step 3**

One has

(20) $$\begin{array}{c} b^{k+1}=b^{k}+\left(\nabla u^{k+1}-d^{k+1}\right). \end{array}$$

Step 2 can be solved by the method in Section 2.1 and Step 3 can be solved directly. To solve Step 1, we use gradient descent method

(21) $$\begin{array}{c} u^{k+1}=\underset{u}{\text{argmin}}\lambda {||b-Au||}_{2}^{2}+\mu {||d^{k}-\nabla u-b^{k}||}_{2}^{2}. \end{array}$$

Equation (21) is derivable and derivation of L_2_ regularization as follows:

(22) $$\begin{array}{c} g\left(u\right)={||b-Au||}_{2}^{2},\;\;\frac{\partial g\left(u\right)}{\partial u}=2A^{T}\left(b-Au\right). \end{array}$$

To derivate (21), we have

(23) $$\begin{array}{c} g^{k}=2\lambda A^{T}\left(Au-b\right)+\mu {\left[{||d^{k}-\nabla u-b^{k}||}_{2}^{2}\right]}^{\prime }. \end{array}$$

Then

(24) $$\begin{array}{c} u^{k+1}=u^{k}-\alpha g^{k}, \end{array}$$

where *k* represents iteration numbers and parameter *α* can be acquired by the following equation:

(25) $$\begin{array}{c} \alpha^{k+1}=\min\left(\alpha^{k},\beta \times {||u^{k}-u^{k+1}||}_{2}^{2}\right). \end{array}$$

## 3. Experimental Study

### 3.1. Numerical Simulation

**Figure 2:**
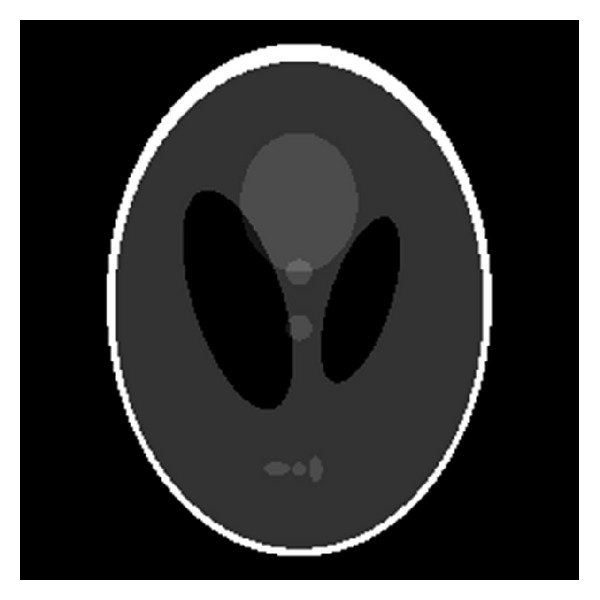
Shepp-Logan phantom.

In this section, we study the ART algorithm, TV based ART algorithm (ART-TV), and L_1/2_ regularization based Split Bregman method (SpBr-L_1/2_) and analyze the reconstructed images. In this paper, we test Shepp-Logan phantom as shown in Figure 2, and the size of phantom image is 256 × 256. We assume that the CT system was viewed as a typical parallel-beam geometry, and the scanning range was from 0° to 180° with a *θ* angular increment; projection angles can be indicated as

(26) $$\begin{array}{c} \theta_{i}=180\times \frac{\left(i-1\right)}{N_{\text{view}}},\;i=1,2,\ldots ,N_{\text{view}}, \end{array}$$

where *N*_view_ is the number of projection angles.

**Figure 3:**
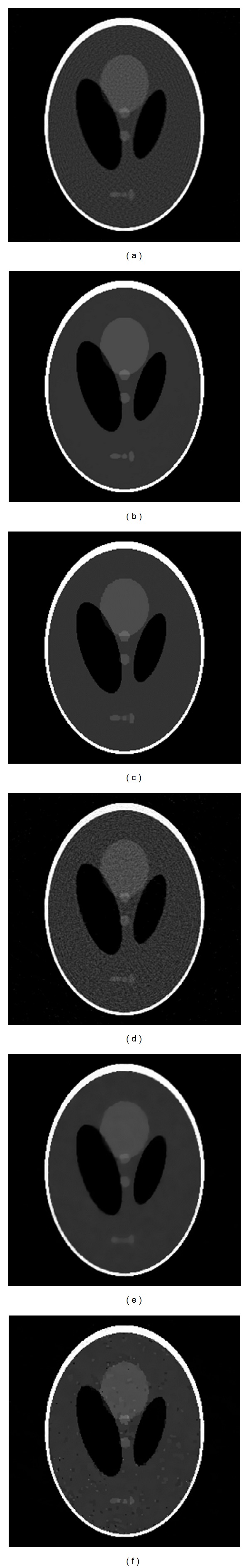
The reconstructed images using three different reconstruction algorithms from the noise-free and noise data. (a)–(c) Reconstructed images from noise-free data: (a) reconstructed image using ART method, (b) reconstructed image using ART-TV method, and (c) reconstructed image using SpBr-L_1/2_ method; (d)–(f) reconstructed images from noise data: (d) reconstructed image using ART method, (e) reconstructed image using ART-TV method, and (f) reconstructed image using SpBr-L_1/2_ method.

We will compare the reconstruction results from noise-free and noise data and projection numbers *N*_view_ = 60. In the simulation, we add 0.01% Gaussian noise to noise-free projection data, and iteration number for every reconstruction algorithm is 50; the reconstruction results are shown in Figure 3.

**Figure 4:**
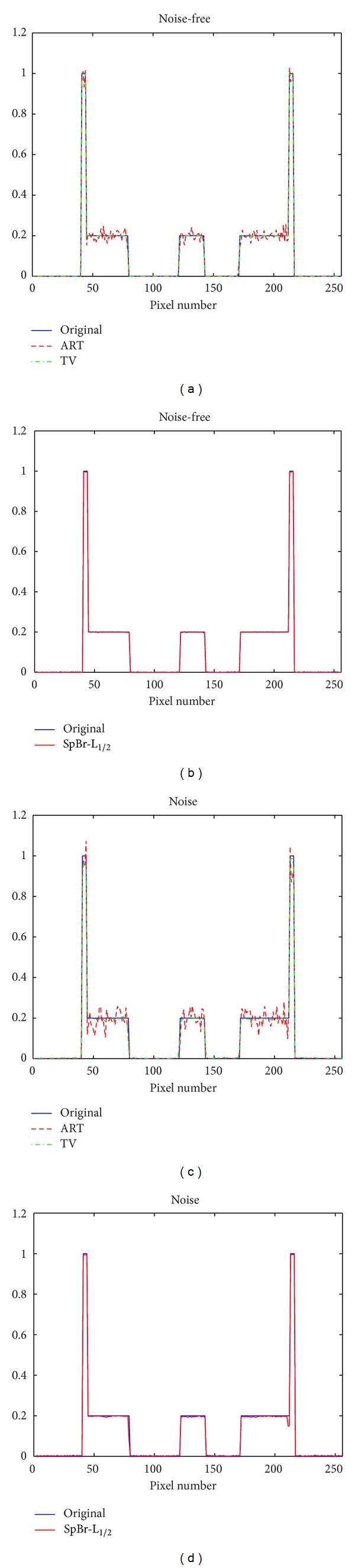
The comparison between reconstructed images using three different reconstruction algorithms and original Shepp-Logan image. (a) The profiles of line 128 in reconstructed images using ART and ART-TV methods from noise-free data and original Shepp-Logan image, (b) the profiles of line 128 in reconstructed images using SpBr-L_1/2_ methods from noise-free data and original Shepp-Logan image, (c) the profiles of line 128 in reconstructed images using ART and ART-TV methods from noise data and original Shepp-Logan image, and (d) the profiles of line 128 in reconstructed images using SpBr-L_1/2_ methods from noise data and original Shepp-Logan image.

From Figures 3 and 4, we can see that the reconstructed images using ART and ART-TV methods contain a lot of noise and artifacts from noise-free and noise data, while the reconstructed images using SpBr-L_1/2_ method include less noise and artifacts and have clearer edges.

In order to evaluate the quality of reconstructed images, we use root mean square errors (RMSE) as the evaluation index. The definition of RMSE is

(27) $$\begin{array}{c} \text{RMSE}=\sqrt{\frac{\sum_{i=1}^{M}\sum_{j=1}^{N}{\left(u_{ij}-u_{ij}^{*}\right)}^{2}}{M\times N}}, \end{array}$$

where *u* and u* are the reconstructed image and original image, respectively, and the image size is *M* × *N*.

As shown in Table 1, the RMSE of reconstructed image using SpBr-L_1/2_ method is much smaller than reconstructed images using ART and ART-TV methods, which means SpBr-L_1/2_ can reconstruct higher-quality images.

**Figure 5:**
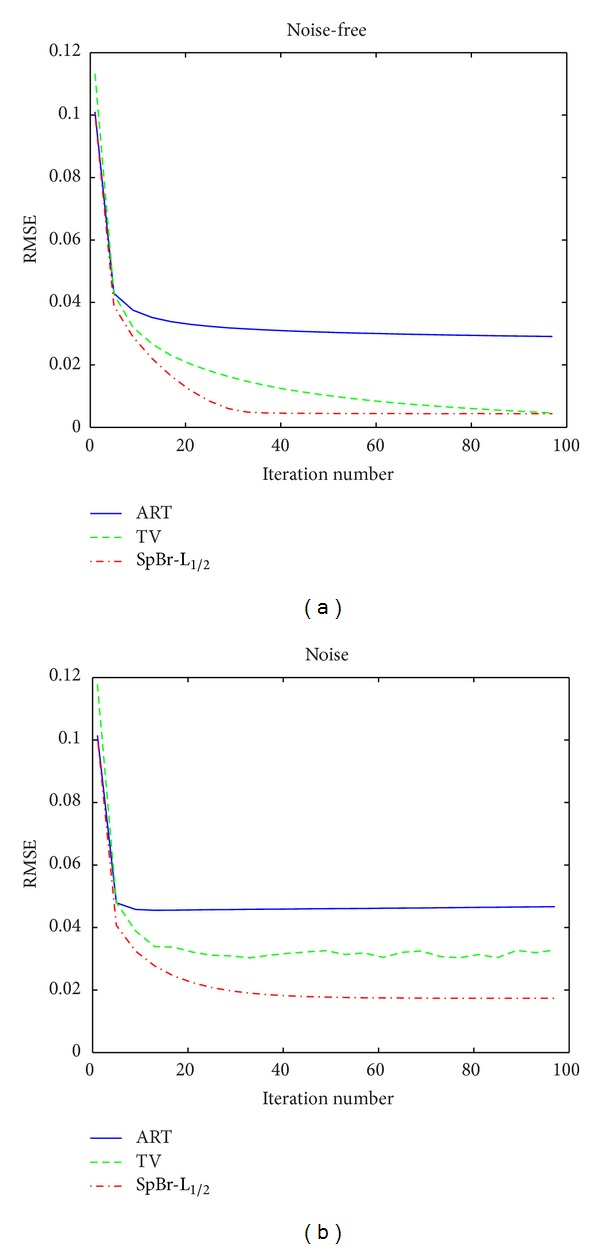
The RMSE line of reconstructed images with different reconstruction algorithms at 60 projection angles and different iteration numbers, and the iteration numbers range from 1 to 100. (a) The RMSE of reconstructed images from noise-free projection data and (b) the RMSE of reconstructed images from noise projection data.

**Figure 6:**
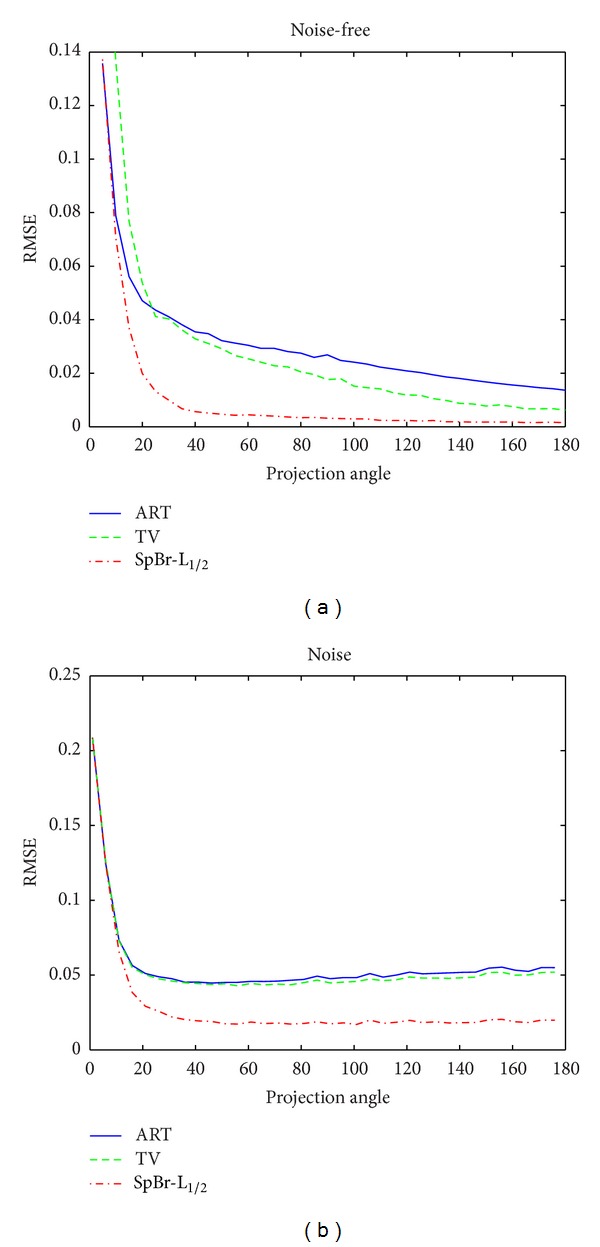
The RMSE line of reconstructed images with different reconstruction algorithms at 50 iteration numbers and different projection angles; the projection angles range from 1 to 180. (a) The RMSE of reconstructed images from noise-free projection data and (b) the RMSE of reconstructed images from noise projection data.

As shown in Figure 5, the SpBr-L_1/2_ algorithm can reconstruct high-quality images at less iteration numbers, which indicates that SpBr-L_1/2_ algorithm can accelerate iterative convergence. From Figure 6, we can see that SpBr-L_1/2_ algorithm can reconstruct high-quality images at less projection numbers.

### 3.2. Real Data Study

**Figure 7:**
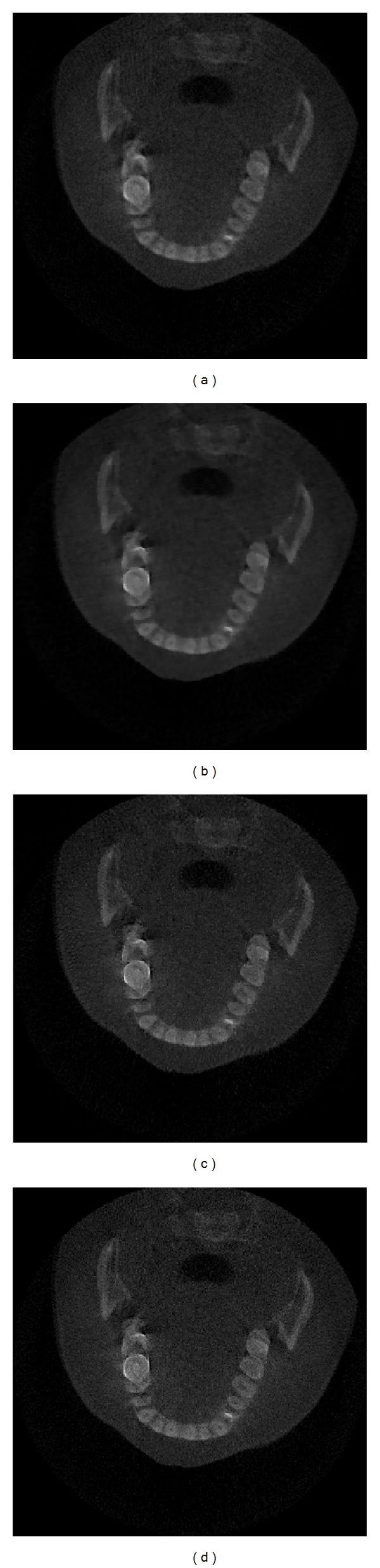
The reconstructed images using three algorithms from real projection data; iteration numbers are 50. (a) Reconstructed image using ART with original projection data, (b) the reconstructed image using ART method, (c) the reconstructed image using ART-TV method, and (d) the reconstructed image using SpBr-L_1/2_ method.

In this section, we reconstruct oral images using three different algorithms with real projection data, where projection numbers are 90 and iteration numbers are 50 while the original projection numbers are 360. And as shown in Figure 7(a), we evaluate three images with reconstructed image using ART with original projection data as a standard image. The reconstructed images and RMSE of three reconstructed images are shown in Figure 7 and Table 2.

As shown in Figure 7, the reconstructed images using ART and ART-TV method have more noise and artifacts, while the reconstructed image using SpBr-L_1/2_ method has less noise and artifacts. And from Table 2, the RMSE of reconstructed image using SpBr-L_1/2_ method is smaller than that of reconstructed images using ART and ART-TV methods, which means SpBr-L_1/2_ can reconstruct higher-quality images with clearer details. From the comparison of reconstructed images using different algorithms, we can see that SpBr-L_1/2_ can reconstruct high-quality images at less projection angles and less iteration numbers.

## 4. Discussions and Conclusion

There are several issues worth further discussion in the reconstruction study. Firstly, the thresholding algorithm was not applied to solve the L_1/2_ regularization problem. There are two reasons. First, if the thresholding algorithm is applied to solve the L_1_ regularization problem, the edge and details of reconstructed images will not be clear, while the SpBr-L_1/2_ without thresholding algorithm reconstructs images with clear edges. Then, if the threshold value is settled to solve L_1/2_ regularization problem, the reconstruction speed will be reduced a lot. Secondly, there are some artifacts in the reconstructed image using SpBr-L_1/2_  method from noise data. The reason is that L_1/2_ regularization is closer to L_0_ regularization, and the ability of denoising of L_0_ regularization is bad; therefore the ability of denoising of L_1/2_ regularization is not good enough. Thirdly, in the reconstruction from real projection data, the reconstructed image using ART-TV method lost some details, while details of reconstructed image using SpBr-L_1/2_ method are much clearer.

In the further research, we will try to use SpBr-L_1/2_ algorithm in interior CT and study the region of interest (ROI) reconstruction, which will reduce radiation dose as much as possible.

In conclusion, we proposed a CT reconstruction algorithm based on L_1/2_ regularization; the reconstructed results demonstrate that SpBr-L_1/2_ can reconstruct high-quality images from few-view data. In the urgent demand of radiation reduction, SpBr-L_1/2_ algorithm will have great potential in clinical application.

## Figures and Tables

**Table 1:** The RMSE of reconstructed images using three different algorithms from noise-free and noise data.

Methods	ART	ART-TV	SpBr-L_1/2_
Noise-free	0.0305	0.0104	0.0044
Noise	0.0388	0.0274	0.0102

**Table 2:** The RMSE of reconstructed images using three different algorithms with real projection data.

Methods	ART	ART-TV	SpBr-L_1/2_
RMSE	0.0270	0.0256	0.0236
